# Selecting Representative Samples From Complex Biological Datasets Using K-Medoids Clustering

**DOI:** 10.3389/fgene.2022.954024

**Published:** 2022-07-18

**Authors:** Lei Li, Linda Yu-Ling Lan, Lei Huang, Congting Ye, Jorge Andrade, Patrick C. Wilson

**Affiliations:** ^1^ University of Chicago Department of Medicine, Section of Rheumatology, University of Chicago, Chicago, IL, United States; ^2^ Knapp Center for Lupus and Immunology Research, University of Chicago, Chicago, IL, United States; ^3^ Center for Research Informatics, University of Chicago, Chicago, IL, United States; ^4^ Key Laboratory of the Ministry of Education for Coastal and Wetland Ecosystems, College of the Environment and Ecology, Xiamen University, Xiamen, China; ^5^ Department of Pediatrics, University of Chicago, Chicago, IL, United States

**Keywords:** single cell, sampling, k-medoids, R, antibody candidate selection

## Abstract

Rapid growth of single-cell sequencing techniques enables researchers to investigate almost millions of cells with diverse properties in a single experiment. Meanwhile, it also presents great challenges for selecting representative samples from massive single-cell populations for further experimental characterization, which requires a robust and compact sampling with balancing diverse properties of different priority levels. The conventional sampling methods fail to generate representative and generalizable subsets from a massive single-cell population or more complicated ensembles. Here, we present a toolkit called Cookie which can efficiently select out the most representative samples from a massive single-cell population with diverse properties. This method quantifies the relationships/similarities among samples using their Manhattan distances by vectorizing all given properties and then determines an appropriate sample size by evaluating the coverage of key properties from multiple candidate sizes, following by a k-medoids clustering to group samples into several clusters and selects centers from each cluster as the most representatives. Comparison of Cookie with conventional sampling methods using a single-cell atlas dataset, epidemiology surveillance data, and a simulated dataset shows the high efficacy, efficiency, and flexibly of Cookie. The Cookie toolkit is implemented in R and is freely available at https://wilsonimmunologylab.github.io/Cookie/.

## Introduction

Single-cell sequencing techniques grew extensively by developing higher cell throughput, improved sensitivity, better reliability, and more modalities in the last decade (Tang et al., 2009; Peterson et al., 2017; Svensson et al., 2018; Stuart and Satija, 2019). Among all biological topics and contexts, the immune system contains a massive amount of highly diverse cells in phenotype and function, and therefore has benefited enormously from the application of novel single-cell RNA sequencing (scRNA-seq) in order to investigate the development and activation of immune cells (Bendall et al., 2014; Goldstein et al., 2019; Winkels et al., 2018; Zhang et al., 2019). In detail, people are able to characterize diverse properties, for example, transcriptome expression, B cell repertoire (BCR), and surface protein expression, for a massive amount of single immune cells in a single experiment (Peterson et al., 2017; Goldstein et al., 2019; Li et al., 2021). This gives people immense power to comprehensively scan a whole population of immune cells in order to identify candidates for further experimental characterization (e.g., neutralizing and antibody binding) (Dugan et al., 2021). Since experimental characterizations are usually resource and human labor intensive, the number of candidates is usually limited by budget. Therefore, a sampling strategy that is capable to effectively select compact and representative samples from a massive population with diverse properties is highly demanded.

The selection of representative samples to reflect the properties and maxima proportion of a large population is a common problem (McCarty et al., 1997; Tominaga, 1998; Siddiqui et al., 2006; Chen et al., 2016). Compared to conventional sampling problems, it imposes even more challenges when selecting samples from a massive biological dataset, for example, single-cell atlas dataset, as biological sample selections are often size sensitive and have diverse properties with different types and importance, and all properties need to be balanced in the selection. More specifically, novel biological data, represented by single-cell atlas data, proposed three specific requirements to representative sampling. First, the selected samples should be able to maximally represent the distribution of original population. Second, the sample size should be as compact as possible in order to save human labor and reagents. Third, randomness of selected samples is not preferred in those cases because subsequent experimental design requires robust and repeatable results. In general, a sampling strategy that can achieve the balance between scientific sufficiency and expense economy with high efficiency is preferred, which can effectively address the contradiction between growing detection capabilities and limited experimental capabilities.

Sampling from a large population has been well studied, and multiple probability and nonprobability sampling methods, including simple random sampling, systematic sampling, cluster sampling, stratified sampling, quota sampling, and snowball sampling, have been proposed for practical sampling problems (Cochran, 2007; Fricker, 2008). Two implementations of probability sampling methods, R package “sampling” and “survey,” have been developed and widely used in the community (Tillé and Matei, 2006; Tillé et al., 2016; Lumley and Lumley, 2019). Those conventional methods do not or rarely use data structure in sampling; therefore, they fail to maximally balance the given properties. Some minor groups maybe ignored causing samples in those groups being rejected. Furthermore, the randomness in the results of probability sampling methods is not preferred or even strictly prohibited in candidates sampling of single-cell atlas data and some other contexts (e.g., influenza surveillance) because robust and repeatable results of each step are crucial for these studies. In addition, a group of Markov chain Monte Carlo (MCMC)–based sampling methods, for example, Metropolis–Hastings sampling and Gibbs Sampling, were proposed to solve sampling problem from high-dimensional population (Geman and GemanHastings, 1970;, 1984). These MCMC-based methods select samples by using data distribution on multiple properties of whole population, and therefore can generate much more representative results than conventional sampling methods. However, MCMC-based sampling methods are usually used to estimate parameters of unknown distribution by constructing a big stochastic process from a given population, or to generate representative samples from a known probability distribution. For single-cell datasets, the joint probability distributions of multiple properties are usually unknown and incalculable, which makes MCMC-based sampling unavailable. Moreover, after algorithms reach a convergence, MCMC-based methods prefer to select more samples for better estimation, which contradicts the requirement of compatibility of single-cell data selection. In practice, compatibility, stability, and representativeness on massive population are three priorities that may not be easily achieved by existing sampling methods. Meanwhile, a systematic approach to determine an appropriate sample size is required.

To overcome these challenges, we developed a k-medoids clustering-based sampling strategy. This method achieves both stable and representative results and allows users to determine an optimized sample size by evaluating the coverage of key properties. We have made Cookie available on a public repository for users worldwide: https://wilsonimmunologylab.github.io/Cookie/.

## Materials and Methods

### Datasets

**Simulated dataset:** We generated a simulated dataset with 10,000 samples and five factors. We generated three-character type factors (Factors 1–3) and two numerical type factors (Factors 4 and 5). Factor 1 is a character factor with levels from 1—20; Factor 2 is a character factor with levels from 1—50; Factor 3 is a character factor with levels from group 1—group 9; Factor 4 is a numerical factor with integer values within the range of 1–20; and Factor 5 is a numerical factor with floating number values that follow a normal distribution (mean = 0, standard deviation = 1). There are a total of 10,000 records in this dataset, and we also extract different size subsets (1,000, 2,500, and 5,000) from this dataset to test the efficiency of our method on different data sizes.

**Single-cell B cell dataset:** In the vaccine clinical trial, we applied Cookie to unbiasedly select representative monoclonal antibodies for expression/characterization from 1,937 antibodies from 19 subjects, seven transcriptional clusters, four isotypes (IgA, IgG, IgM, and IgD), various V locus gene usages, and various CDR3 peptide lengths. We generated these monoclonal antibodies using single-cell B cell receptor cloning of a pair of the heavy chain and light chain genes followed by *in vitro* expression to further characterize mAb specificity and function to evaluate the vaccine response.

**Human influenza H1N1 surveillance viral sample dataset:** We downloaded all data records of human influenza H1N1 viruses collected between August 1, 2018 and August 1, 2019 from GISAID database (https://www.gisaid.org/) (Shu and McCauley, 2017). A total of 8,449 viruses were retained after removing the redundant records. By comparing the sequences to the WHO recommended H1N1 vaccine strain A/Michigan/45/2015 (H1N1) (https://www.cdc.gov/flu/season/flu-season-2018-2019.htm), we calculated mutation numbers of the HA1 protein for all H1N1 viruses. Mutation numbers of H1 epitopes was also calculated for all H1N1 viruses. The protein sequences were aligned using MAFFT v7.427 (Katoh and Standley, 2013). Positions of five epitopes of H1 protein were adopted from the literature (Li et al., 2020). In this dataset, there are four factors: month, continent, mutations, and mutations on epitopes. The month factor is a character factor with 12 levels (2018-08 to 2019-07); the continent factor is a character factor with six levels (Africa, Asia, Europe, North America, Oceania, and South America); mutations is a numerical factor with integer values within the range of 0–14; and mutation on epitope is a numerical factor with integer values within the range of 0–3. The dataset was downloaded on August 29, 2019.

### Data Vectorization

Each sample was represented by a vector, and dimensions of the vector are factors from the original data (e.g., subject, transcriptional cluster or just “cluster,” gender, and antibody isotype). All factors can be divided into two groups: character factors and numerical factors. Character factors usually have multiple (two or more) discrete values, representing clusters, subjects, groups, batches, and so on. Numerical factors have continuous numerical values with either integers or floating numbers, and different levels can be quantified by the difference of these values. To clarify, character factors also have numerical levels. The difference between numerical factors and character factors is that the levels of character factors are none-quantifiable labels and the levels of numerical factors are quantifiable values. The difference between any two levels can be quantified by the difference of their values. For example, for a character factor (e.g., a cluster) which has three levels, the difference between levels 1 and 2 is equal to that between levels 1 and 3. For a numerical factor (e.g., number of mutations) which has three levels, the difference between levels 1 and 2 is smaller than that between levels 1 and 3.

### Linearization

In biological datasets, logarithmic values are a commonly used data type (e.g., HI titers in Influenza hemagglutination inhibition assays, https://www.cdc.gov/flu/about/professionals/antigenic.htm). In order to compare values within a factor, values of all the numerical factors should be linear (Sun et al., 2013). All of the nonlinear factors should be linearized in advance of further normalization. A logarithm will transfer logarithmic values into linear values. Users should choose the base number of the logarithm according to their dataset. For example, original HI titers are equal to $HI=10\times 2^{n}$ , where n is the number of dilutions, so the base of logarithm will be two for HI data; thus, the linearized HI titer should be: $HI\prime =log_{2}\left(HI/10\right)$ .

### Normalization

Data normalization is essential for numerical factors in order to be comparable with other factors (Hancock et al., 1988; Singh and Singh, 2020). Here, we adopted a min–max normalization method to scale a numerical factor such that all values are within the range of [0,1]. The normalized value x′ can be calculated by the following equation:

$$x\prime =\frac{x-x_{min}}{x_{max}-x_{min}},$$

where x , $\;x_{min}$ , and $x_{max}\;$ are the original values, the minimum value of original samples, and the maximum value of original samples, respectively.

### Distance Calculation

For any two samples, we calculated pairwise distances following a two-step strategy, that is, (1) compute the differences between two samples on individual factors and 2) calculate the overall distance by integrating the differences from all factors. We applied binary distance coding to represent the difference among character factors (0 for equal and 1 for difference). For numerical factors, the distance is equal to the absolute value of the difference. Then, overall distance D was calculated by integrating differences on all factors $d_{i}$ using a weighted $L_{1}$ norm (summary of their absolute values with weights). $w_{i}$ denotes weight of the i -th factor:

$$D=w_{i}d_{i1}=\overset{n}{\underset{i=1}{\sum }}|w_{i}d_{i}|.$$

In case of missing values in the dataset, we consider the difference between missing values and any other value as 0. This strategy prevents introducing biases from comparing missing values with real values.

### Embedding

To visualize the sampling results, we utilized two state-of-the-art nonlinear dimensional reduction methods, that is, uniform manifold approximation and projection (UMAP) and t-distributed stochastic neighbor embedding (t-SNE) (McInnes et al., 2018; Van der Maaten and Hinton, 2012). Both embedding methods accept pairwise distances as input and render a 2D projection of the samples.

### Roles of Factors

In this workflow, we designed three different roles for factors which are as follows: prime factor, important factors, and regular factors. All factors contribute to the distance calculation. The prime factor and important factor are optional in a sampling. The prime factor is unique in a dataset, and the representative samples were selected evenly from each element of prime factor (e.g., subject and animal) instead of selecting from the entire dataset. Important factor indicates a 100% coverage requirement and can be multiple. Regular factors contribute to the distance calculation as other factors do but without any specific requirement to the sampling. The determination of prime factor and important factor is up to users own choice. Users can determine each factor from their dataset to any role (prime, important, or regular) according to their sampling needs and domain knowledge. For example, in the sampling from our single-cell B cell dataset, we would like to select samples from each subject (donors), so that “subject” was set as the prime factor. We would also like to investigate all transcriptional clusters, so that “cluster” was set as an important factor. The rest of the factors were set as regular factors.

### Clustering-Based Two-step Sampling Strategy

To achieve a high sampling coverage with good representativeness, we designed a two-step sampling strategy. The first step is to select N samples from the entire dataset or from each subject if the prime factor was set using a k-medoids clustering method. The cost function in k-medoids algorithm is given as (Kaufman et al., 1987)

$$c=\underset{C_{i}}{\sum }\underset{P_{i}\in C_{i}}{\sum }|P_{i}-C_{i}|,$$

where $C_{i}$ denotes the medoid and $P_{i}$ denotes the sample. The most common implementation of k-medoids clustering is the partitioning around medoids (PAM) algorithm. More specifically, samples can be clustered into multiple evenly distributed clusters using the k-medoids clustering method. The medoid for each cluster can be considered as the most representative samples of the corresponding cluster. This step guarantees the representativeness of selected samples.

The second step is to investigate the coverage rate of all important factors (defined by users) from the representative candidates picked by k-medoids clustering from last step. If any important factor has a coverage rate lower than 100%, then an additional selection will be performed to pick the proper samples from the unpicked population to cover all the levels/categories of the important factor. The strategy for adding qualified samples is as follows: for a category of an important factor that has not been covered by samples selected in step 1, if there is more than one candidate, we select the one that has the largest local distances with all selected samples in the first step. We define local distance as

$$D_{Local}=\underset{i\in S}{\min}D_{i},$$

where $D_{i}$ denotes the distance between the current sample and the i -th selected samples. S denotes the set of selected samples.

### Evaluation of Sampling

The quality of sampling can be evaluated and quantified by coverage rate on each single factor. Here, for character factors, we define the coverage rate as

$$\text{Coverage rate }=\frac{number\;of\;levels\;in\;selected\;samples}{number\;of\;levels\;in\;the\;original\;population}.$$

To be consistent, for numerical factors, since they have been scaled into [0,1], we assigned them to ten evenly divided bins ([0, 0.1] [0.1, 0.2], … [0.9, 1]); and then the coverage rate of numerical factors can be defined as

$$\text{Coverage rate }=\frac{number\;of\;levels\;in\;selected\;samples}{number\;of\;levels\;in\;the\;original\;population}.$$

Of note, a statistical test between original population and selected population can also be used to evaluate the sampling quality for a numerical factor.

Using the quantified coverage rate on each single factor, users can determine an optimized sample size that balances both factor coverage and cost.

Users can also check the distribution of selected samples on each factor. For example, if the distribution of selected samples is identical to that of the original samples, it indicates that the sampling is of high quality. The similarity of distribution on each factor between the original population and selected samples can also be approximately quantified by Pearson correlation coefficient.

$$r_{xy}=\frac{\sum_{i=1}^{n}\left(x_{i}-\bar{x}\right)\left(y_{i}-\bar{y}\right)}{\sqrt{\sum_{i=1}^{n}{\left(x_{i}-\bar{x}\right)}^{2}}\sqrt{\sum_{i=1}^{n}{\left(y_{i}-\bar{y}\right)}^{2}}},$$

where n is the number of levels in this factor and $x_{i}$ and $y_{i}$ are the number of samples of the i -th level of this factor for two sets of samples; $\bar{x}=\frac{1}{2}\overset{n}{\underset{i=1}{\sum }}x_{i}$ and analogously for $\bar{y}$ .

## Results

### Cookie: Representative Sample Selection From a Massive Population Using K-Medoids Clustering

**FIGURE 1:**
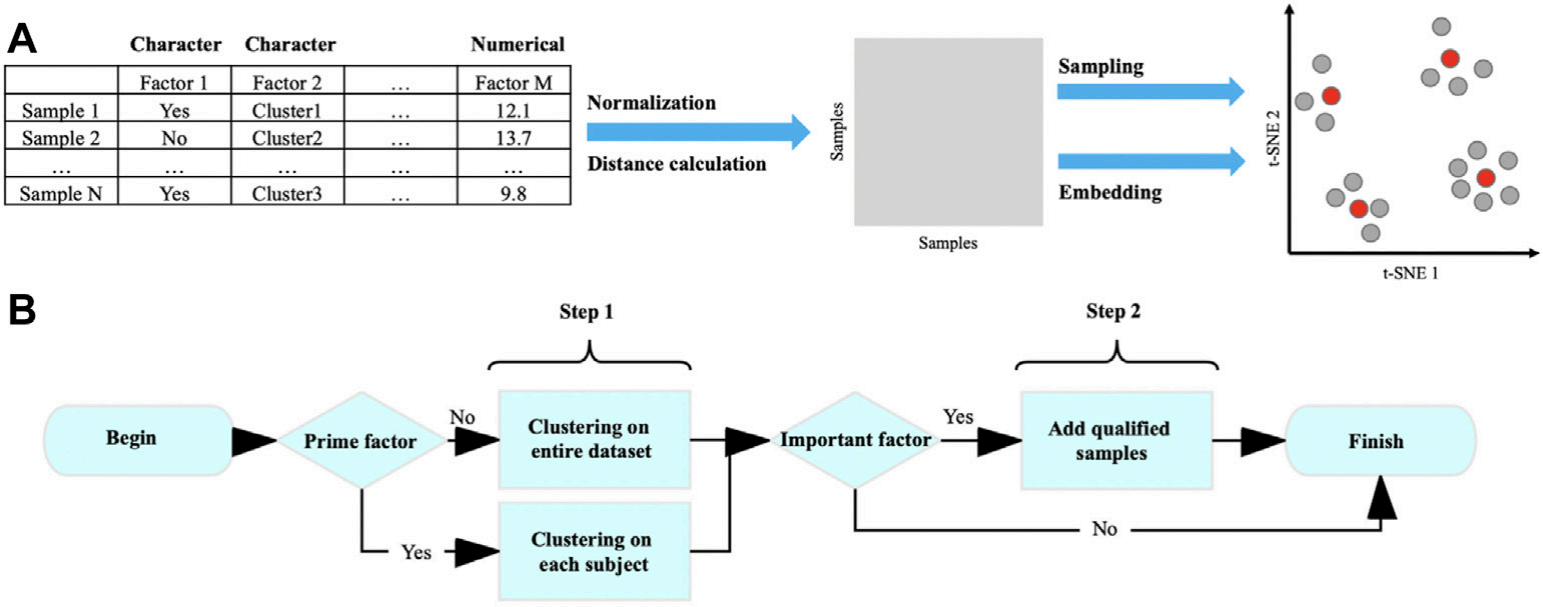
Workflow of k-medoids–based sampling. **(A)** Workflow of Cookie pipeline and **(B)** selecting representative samples using k-medoid clustering method.

Here, we present Cookie, a user-friendly toolkit, to select representative samples from massive populations (especially single-cell sequencing data). The prime idea of this method is quantifying and vectorizing all samples in order to quantify their dissimilarity by their Manhattan distances, and then samples can be classified into several clusters by k-medoids clustering according to their dissimilarity and centers of those clusters are representative samples (Figure 1). In detail, each sample is presented as a numerical vector, and the elements of the vector are attributes of the original data (e.g., subject, transcriptional cluster, and gender). The relationships/dissimilarity among all samples were quantified by calculating a pairwise Manhattan distance matrix. Based on that, a two-step sampling strategy was performed as follows: 1) classify samples into k clusters by k-medoids clustering and select centers of the k clusters and 2) add proper samples to qualify the coverage requirement on specified factors. This method is composed of four steps: normalization, distance calculation, sampling, and embedding (Figure 1A). In this toolkit, we defined three roles of factors, prime, important, and regular, to help users better describe their sampling goal. To achieve better representativeness, we designed a two-step sampling strategy (Figure 1B). The first step is to select k samples using k-medoids method from the entire population or from each subject of prime factor (Kaufman et al., 1987; Schubert and Rousseeuw, 2018). The second step is to add qualified samples to cover all the categories/levels of important factors (see *Results* for details). Cookie calculated the summary of distances between candidates and selected samples and always picks the one with largest distance if there is more than one candidate.

### Application of Cookie on Single-Cell Atlas Data to Select Candidates of Monoclonal Antibody for Further Experimental Characterization

**FIGURE 2:**
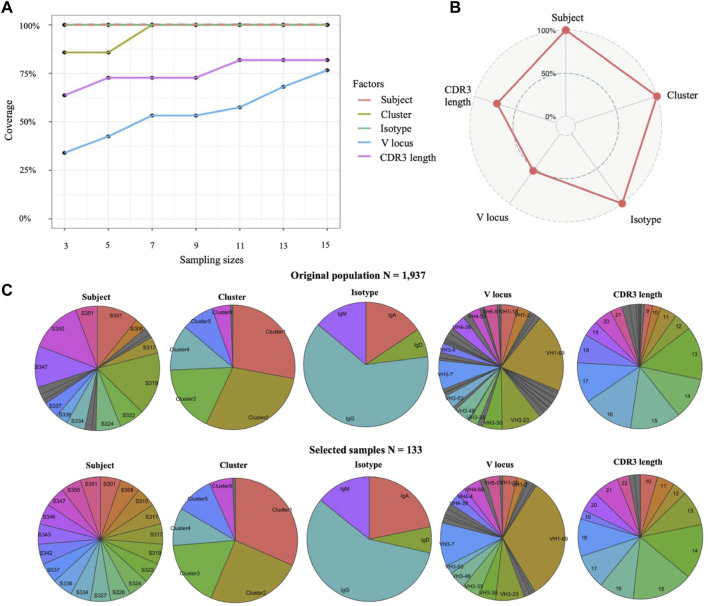
Select representative samples from a large single-cell population. **(A)** Determine appropriate sample size by quantifying coverage on all factors. Line of subject factor was indicated by dash line to avoid overlap. **(B)** Coverage on each factor of selected samples. **(C)** Compare distributions on each factor between original population and select samples.

We applied this method to select candidates of monoclonal antibody from the isolated genes for 1,937 antibodies for laborious protein expression and downstream analysis. The single-cell atlas dataset consists of seven transcriptional clusters, 19 subjects, a variety of V gene usages, four major isotypes, and a variety of complementarity-determining region 3 (CDR3) lengths among the genes for 1,937 antibodies. Our goal was to 1) select representative samples for laborious protein expression from the genes of 1,937 antibodies and 2) determine the optimized sample size that can balance sampling coverage on all factors and economy. For this dataset, we wanted to evenly select samples from each subject, and a 100% coverage is required for transcriptional clusters. We set “Subject” as the prime factor and “transcriptional cluster” as an important factor. As shown in Figure 2A, coverage of all factors is positively correlated with sample sizes from each subject, and N = 7 is the optimal sample size from each subject because 100% coverage of three key factors (subject, cluster, and isotype) and high coverage of other two factors have been achieved. After determining the sample size to seven per subject, we selected 133 samples from 1,937 antibodies with a 100% coverage on subject, cell cluster, and isotype (Figure 2B). We observed highly similar distributions between selected 133 samples and the original population by comparing the distribution of five factors (Figure 2C). Moreover, the total runtime of sample size determination and sampling is less than ten seconds. In conclusion, results on real single-cell B cell dataset showed that Cookie toolkit is effective and efficient in selecting candidate antibodies for further experimental characterization from massive single-cell population.

### Application of Cookie on Human Influenza Virus Surveillance Data

**FIGURE 3:**
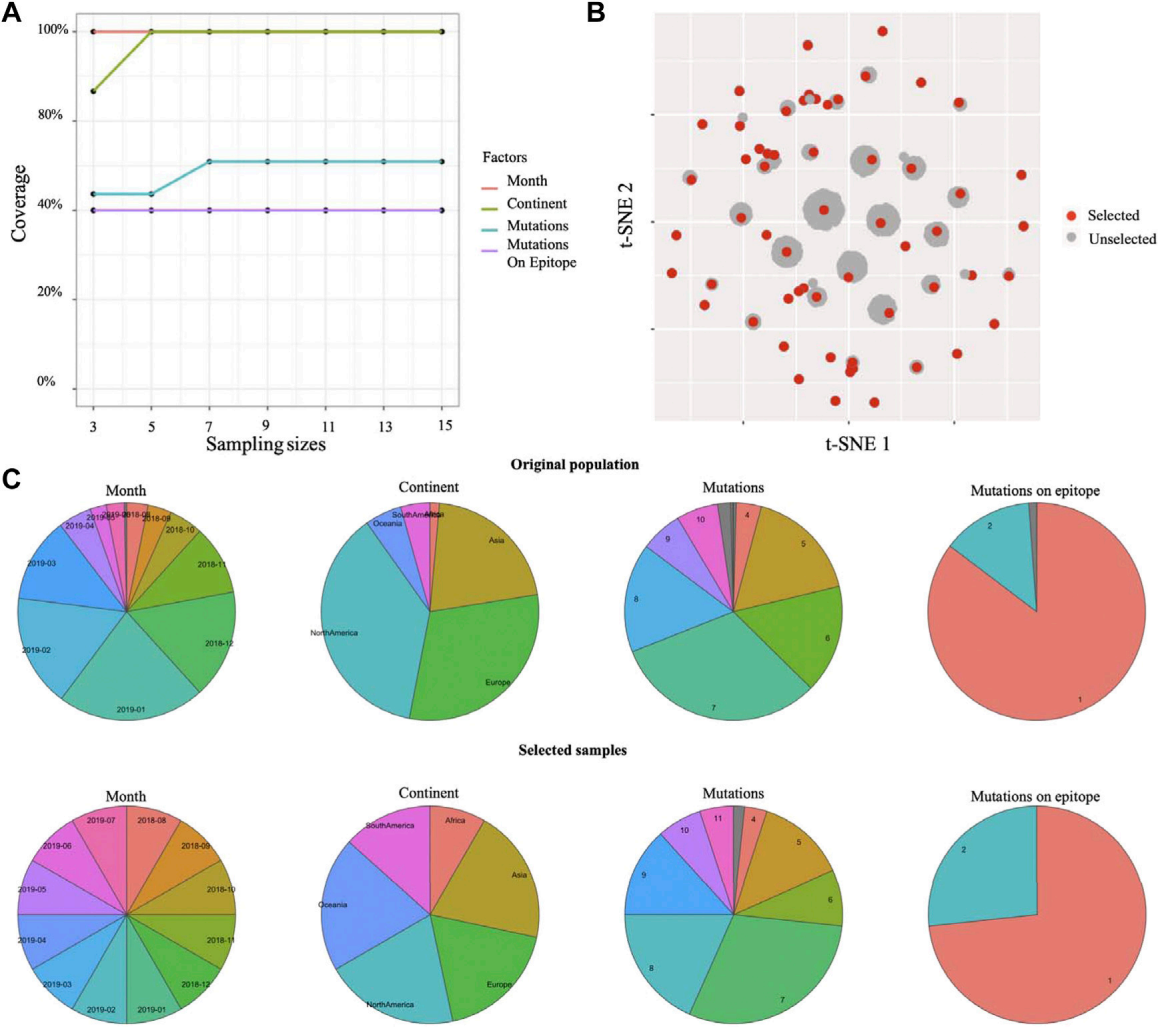
Selected representative samples from human Influenza H1N1 surveillance data. **(A)** Testing coverage rate on each factor of different sample sizes. **(B)** Selected samples and unselected samples on 2D visualization (t-SNE). **(C)** Distributions of each factor of original population and selected samples.

Beside single-cell sequencing data, Cookie toolkit is also compatible with more biological applications. Here, we examined the flexibility of our method on a different type of biological dataset. Influenza virus has a highly mutable replication process allowing it to escape from immunity, often on an annual basis (Kosikova et al., 2018). In order to control this escape, each influenza season, tens of thousands of samples of influenza viruses are collected from surveillance programs across six continents (Lackenby et al., 2018). Identifying antigenic variants from those viral samples is the key to a successful vaccine strain selection to generate a vaccine protective against the most common viral variants (Koel et al., 2013). The main challenge is that people can only investigate antigenic profiles for a small proportion of all viral samples using HI assay, which is time- and labor-intensive. An efficient sampling method that can balance samples with genetic variations, locations, and times of sampling (month) is required. The k-medoids sampling method proposed in this study is capable of addressing this problem. We performed the k-medoids sampling on a human H1N1 influenza dataset with 8,449 viral samples (see dataset section for details) using Cookie toolkit. To identify the earliest antigenic variant, we set “Month” as a prime factor to balance samples from different time periods. The sample size test indicates that a sample size of five (setting sample size to seven will slightly increase the coverage of mutation, if budget allows) is an appropriate choice for this dataset (Figure 3A). With the sample size of five, the selected samples covered all the clusters, and therefore are able to represent all of the genetic-temporal-spatial combined variances (Figure 3B). The distribution of each factor also shows that the selected samples have a highly similar distribution as the original population (Figure 3C). In general, results on two real datasets showed that Cookie specializes in solving contradiction between large detective capabilities and limited experimental capabilities and is compatible with multiple biological contexts.

### Evaluation of Cookie Performance Using Simulated Data of Different Population Sizes

To evaluate the efficiency and compatibility of this method, we generated a simulated dataset (see datasets section for details) and tested our method on this simulated dataset. We compared the runtime of our method on four different data sizes: 1,000, 2,500, 5,000, and 10,000 samples. As shown in table 1, with increasing population sizes, the runtime of the four major steps (distance calculation, nonlinear reduction, sample size test, and sampling) increased exponentially. In addition, sampling from the levels of prime factor is much faster than sampling from the entire population, especially for large populations. That is because the runtime complexity of the k-medoids clustering algorithm (also called as PAM algorithm) is $O(k{\left(n-k\right)}^{2})$ , and the runtime is proportional to population size n and cluster number k . A recent study proposed an optimized k-medoids clustering algorithm called FastPAM that reduces the runtime complexity to $O\left(n^{2}\right)$ (Schubert and Rousseeuw, 2018). By adopting the FastPAM algorithm, the runtime was largely reduced (Table 1). Of note, conventional probability sampling methods are much faster than k-medoids sampling because such methods do not (or rarely) use data structure and distribution of the original population as seen in k-medoids. In conclusion, our results indicate that k-medoids sampling is able to effectively and efficiently select representative samples from large populations (Tables 1, 2). These results also demonstrate that sampling from levels of a prime factor or using algorithm acceleration (FastPAM) could significantly reduce the sampling time.

**TABLE 1:** Runtime of major steps of Cookie pipeline on different population sizes. All the tests were performed on a simulated dataset using a 2015 Apple MacBook Pro (Core i5, 2.7GHZ, 8 GB DDR3 memory). N denotes population size.

Processing Step		Runtime (seconds)			
		N = 1,000	N = 2,500	N = 5,000	N = 10,000
Preprocess	Create object	0.001	0.004	0.004	0.05
	Normalization	0.003	0.005	0.012	0.027
	Distance calculation	0.347	1.77	7.375	32.511
	Nonlinear reduction (t-SNE)	6.759	13.731	50.701	142.668
Prime factor mode* (PAM algorithm)	Sample size test	0.279	1.414	9.097	54.703
	Sampling	0.04	0.195	1.523	6.134
Prime factor mode* (FastPAM algorithm)	Sample size test	0.663	1.036	4.276	43.047
	Sampling	0.123	0.154	0.578	3.089
Nonprime factor mode** (PAM algorithm)	Sample size test	26.258	301.05	1750.68	>3,000
	Sampling	3.517	36.251	261.614	>3,000
Non-prime factor mode** (FastPAM algorithm)	Sample size test	4.403	27.656	115.79	598.854
	Sampling	0.493	3.31	13.522	68.22

* A prime factor is determined in this run. Algorithms for k-medoids clustering are indicated in the brackets.

** No prime factor is determined in this run. Algorithms for k-medoids clustering are indicated in the brackets.

**TABLE 2:** Coverage rates of k-medoids sampling on different population sizes. All the tests were performed on a simulated dataset using a 2015 Apple MacBook Pro (Core i5, 2.7GHZ, 8 GB DDR3 memory). N denotes population size. Tests were generated using the Cookie package with the FastPAM method. The sample size for prime factor mode is set to 10 (from each level of prime factor) and that for no-prime factor mode is set to 100.

Factor		Coverage Rate (%)			
		N = 1,000	N = 2,500	N = 5,000	N = 10,000
Prime factor	Factor 1	84.00	82.00	78.00	80.00
	Factor 2	100.00	100.00	100.00	100.00
	Factor 3	100.00	100.00	100.00	100.00
	Factor 4	90.91	100.00	90.91	90.91
	Factor 5	81.82	72.7	72.7	72.73
Nonprime factor	Factor 1	92.00	86.00	88.00	84.00
	Factor 2	100.00	100.00	100.00	100.00
	Factor 3	100.00	100.00	100.00	100.00
	Factor 4	90.91	100.00	90.91	90.91
	Factor 5	81.82	72.73	72.7	72.73

### Comparison With a Conventional Probability Sampling Method

**FIGURE 4:**
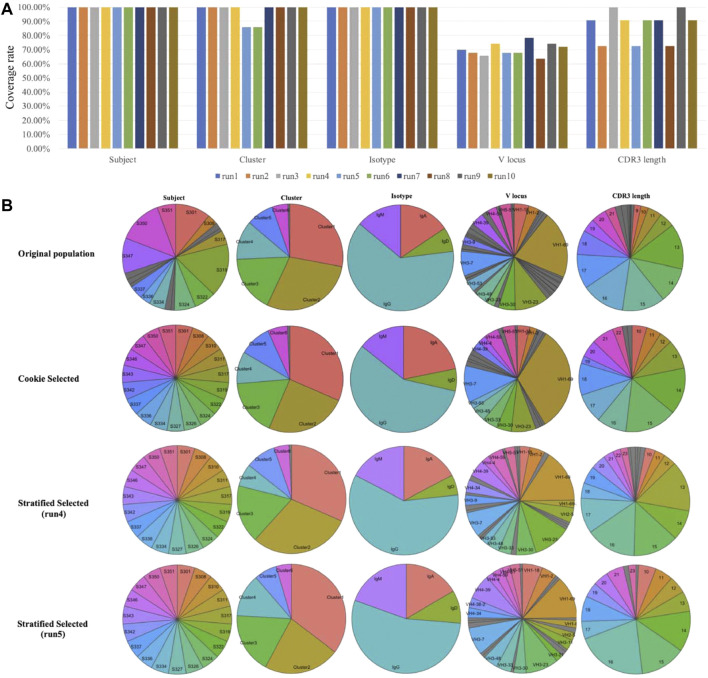
Compare k-medoid sampling with probability sampling method (stratified sampling). **(A)** Coverage rate on each factor of ten independent runs of stratified sampling. **(B)** Distributions of each factor of original population and samples selected by k-medoid sampling and stratified sampling.

The randomness of probability sampling methods is not preferred in antibody selection from single-cell data and some other biological studies. In these cases, distributions and importance of factors are well known. The top priority of sampling is to select the most representative samples based on those factors. Randomness will help less to establish representativeness and may result in inconvenience for further experimental design. Another issue with probability sampling is that the results from two independent probability samplings may be different. Nevertheless, we compared our method to probability sampling methods. We used stratified sampling, the most suitable method for this single-cell dataset among all probability sampling methods, as an example of probability sampling methods. This comparison was performed on our single-cell dataset (see dataset section for details). As shown in Figure 4, we compared our method to the stratified sampling method with the same sampling size (select 133 samples from 1,937 cells). Samples were stratified according to “Subject” in stratified sampling. “Subject” was set as the prime factor and “Cluster” was set as an important factor in our method. We performed ten independent runs of stratified sampling on the single-cell dataset, and the results showed that the coverage rates of each factor among ten runs vary (Figure 4A), with two runs not even covering all cell clusters (run5 and run6). We picked two from the ten runs (run4 the best and run5 the worst) and compared the results to Cookie selection and the original population (Figure 4B). The results show that both k-medoids clustering selection and run4 of stratified sampling are able to represent the original population while run5 fails (fail to select any sample from a small cluster, “Cluster 7”). The results prove that the k-medoids clustering method is not only effective for the selection of representative samples but also able to avoid potential bias caused by the randomness of probability sampling.

## Discussion

Based on a k-medoids clustering strategy, we developed a method to select representative samples from a large population. A similar approach for geographical sampling using a k-means clustering method was developed in a prior study (Walvoort et al., 2010). Their results also proved the representativeness and practicability of application of clustering methods in sampling. Of note, their method requires an existing distance measurement among the original samples. It limited the application range of the method since most of biological/clinical datasets do not satisfy the requirement. By developing a workflow consisting of data vectorization and distance calculation steps, our method normalizes different types of factors into the same scale and quantifies the distances among samples based on those normalized factors. This workflow can quantify relationships among samples for all the populations with multiple numerical and non-numerical factors and greatly expand the range of application of our method. Compared to the previous clustering-based sampling approach, our method is advantageous for single-cell populations with complicated structures (multiple factors with different types and priority levels) and compatible with most of the biological datasets.

Conventional probabilistic/nonprobabilistic sampling methods do not or rarely use data structure in sampling. While it highly improves efficiency of sampling process by not using data structure however, the representativeness of samples through random selections usually cannot be guaranteed. By contrast, our method uses the entire data structure when selecting samples. It generates pairwise distance matrix by considering all factors with different priority levels to quantify relationships among samples. Then our method selects samples using k-medoids clustering method by dividing entire population into k clusters. Since the clustering results are subject to pairwise distance that considers all factors, factors of selected samples are therefore maximumly balanced. In other words, the representativeness of selected samples is achieved by balancing all factors of original population. Of note, considering all details in data structure will result in inefficiency, especially for large populations due to the exponential growth of running time as the sample number increases. By introducing a recent proposed method FastPAM, the runtime complexity was greatly reduced. Simulation results showed that Cookie toolkit is capable for robust and efficient sample selection from large populations.

In practice, the number of candidates to be experimentally characterized is usually limited; therefore, selected sample size should be optimized to balance the representativeness and economy. Conventional methods usually do not offer an effective method to determine an appropriate sample size. Furthermore, the representativeness of a sample selection is usually difficult to evaluate. To overcome this challenge, Cookie implemented coverage rate of factors to quantify and evaluate the representativeness of a sample selection. The method also allows users to determine an appropriate sampling size by comparing coverage rates of different sample sizes. In addition, our framework is highly modularized and extendable; other evaluation metrics, for example, Pearson’s correlation coefficient, can also be incorporated to the evaluation process. By evaluating on different population sizes using both experimental data and simulated data, our method was proven to be effective and efficient.

In conclusion, we proposed a sampling method that achieved representativeness, stability, economy, and universality. The method is implemented in an R package Cookie and is freely accessible on GitHub. We hope this toolkit (package) will help biologists select representative samples in an unbiased manner from large-scale datasets.

### Limitations

There are two major limitations of this workflow. First, there is only one distance metric (Manhattan distance) in current model. Since different distance measurements can highly affect the clustering results, therefore affecting the final sampling results, investigating effects of different distance measurements is promising to improve the clustering and sampling in the future work. The second limitation is that the time complex of calculating the pairwise distance matrix increases exponentially as the sample number increases. It limits the application of this method on future massive datasets (e.g., datasets have more than 50,000 samples). Furthermore, in current model, we approximately quantify the differences between any two levels of a character factor as the same. A more precise strategy for differences quantification of character factors is also needed to improve the sampling results.

### Code Availability

The method is implemented in R and is freely available on GitHub https://github.com/WilsonImmunologyLab/Cookie. The source is also available at Zenodo: https://zenodo.org/record/6639035#.YqdqBRPMIvo. Tutorials and documents are available at https://wilsonimmunologylab.github.io/Cookie/.

The package has been tested under 1) macOS Mojave version 10.14.6 with R version 3.6.0 and RStudio Version 1.2.1335 and 2) ubuntu 18.04 64bit with R version 3.5.

## Data Availability

The original contributions presented in the study are included in the article/Supplementary Material; further inquiries can be directed to the corresponding author.
